# Expression of truncated bile salt-dependent lipase variant in pancreatic pre-neoplastic lesions

**DOI:** 10.18632/oncotarget.11777

**Published:** 2016-09-01

**Authors:** Emmanuelle Martinez, Isabelle Crenon, Françoise Silvy, Jean Del Grande, Alice Mougel, Dolores Barea, Frederic Fina, Jean-Paul Bernard, Mehdi Ouaissi, Dominique Lombardo, Eric Mas

**Affiliations:** ^1^ Aix-Marseille Université, CRO2, Centre de Recherche en Oncologie biologique et Oncopharmacologie, Marseille, France; ^2^ INSERM, UMR_S 911, Marseille, France; ^3^ Assistance Publique Hôpitaux de Marseille, Hôpital de la Timone, Service d'Anatomopathologie, Marseille, France; ^4^ LBM- Assistance Publique Hôpitaux de Marseille, Hôpital Nord, Service de transfert d'Oncologie Biologique, Marseille, France; ^5^ Assistance Publique Hôpitaux de Marseille, Hôpital de la Timone, Service de Gastroentérologie 2, Marseille, France; ^6^ Assistance Publique Hôpitaux de Marseille, Hôpital de la Timone, Service de Chirurgie Digestive et Viscérale, Marseille, France

**Keywords:** bile salt-dependent lipase, pancreatic cancer, antibodies, Abbreviations: BSDL, bile salt-dependent lipase, PDAC, pancreatic adenocarcinoma

## Abstract

Pancreatic adenocarcinoma (PDAC) is a dismal disease. The lack of specific symptoms still leads to a delay in diagnosis followed by death within months for most patients. Exon 11 of the bile salt-dependent lipase (BSDL) gene encoding variable number of tandem repeated (VNTR) sequences has been involved in pancreatic pathologies. We hypothesized that *BSDL* VNTR sequences may be mutated in PDAC. The amplification of *BSDL* VNTR from RNA extracted from pancreatic SOJ-6 cells allowed us to identify a *BSDL* amplicon in which a cytosine residue is inserted in a VNTR sequence. This insertion gives rise to a premature stop codon, resulting in a truncated protein and to a modification of the C-terminal amino-acid sequence; that is PRAAHG instead of PAVIRF. We produced antibodies directed against these sequences and examined pancreatic tissues from patients with PDAC and PanIN. Albeit all tissues were positive to anti-PAVIRF antibodies, 72.2% of patient tissues gave positive reaction with anti-PRAAHG antibodies, particularly in dysplastic areas of the tumor. Neoplastic cells with ductal differentiation were not reactive to anti-PRAAHG antibodies. Some 70% of PanIN tissues were also reactive to anti-PRAAHG antibodies, suggesting that the C insertion occurs early during pancreatic carcinogenesis. Data suggest that anti-PRAAHG antibodies were uniquely reactive with a short isoform of BSDL specifically expressed in pre-neoplastic lesions of the pancreas. The detection of truncated BSDL reactive to antibodies against the PRAAHG C-terminal sequence in pancreatic juice or in pancreatic biopsies may be a new tool in the early diagnosis of PDAC.

## INTRODUCTION

Human bile salt-dependent lipase (BSDL), or carboxyl ester lipase (CEL), is mainly expressed by the acinar cells of the pancreas [1] and the lactating mammary gland [2]. The BSDL or CEL gene is located on band 34.3 of the long arm of chromosome 9 [3, 4]. The gene consists of 11 exons spanning 9884 base pairs (bp). The N-terminal domain of the protein includes bile salt-binding, catalytic and N-glycosylation sites. This domain is encoded for by exons 1 to 10 and is well conserved across evolution [1], whereas exon 11 encodes the C-terminal domain with a region consisting in a variable number of tandem repeats (VNTR). Each repeat consists of 33 bp coding for 11 amino acids [5] with O-glycosylation sites [6], conferring a mucin-like structure to the C-terminal VNTR of BSDL [7]. The six most C-terminal amino-acid residues of BSDL are highly conserved and may play a role in enzyme activity or stability [8]. VNTR can vary slightly in sequence and the total number of repeats differs between alleles. In populations previously investigated, the most frequently detected allele has 16 repeats, which results in a polypeptide made up of 722 amino acids after cleaving of the signal peptide. However, VNTR on exon 11 can range from three to twenty-one repeats, leading to a high level of heterogeneity in protein size both within and between individuals [9, 10]. VNTR also vary according to species, from none in salmon up to 39 repeats in gorilla [11, 12]. It has been suggested that naturally occurring VNTR can influence BSDL function. An association between VNTR length and increased susceptibility to alcoholic pancreatitis has been hypothesized [13]. Furthermore, plasma levels of BSDL and low-density lipoprotein (LDL) correlate positively and indicate a role for BSDL in cardiovascular diseases [14]. Augé et al. [15] demonstrated that BSDL is present within atherosclerotic plaques in arterial walls, and induces smooth muscle cell proliferation, chemotaxic migration of monocytes and oxidized LDL degradation [16–18]. Links between the BSDL gene and diabetes are multiple; circulating antibodies to the C-terminal mucin-like domain of BSDL have been detected in sera of patients with a type-1 diabetes [19], and a single base deletion in either repeat 1 or 4 within the VNTR has been associated with autosomal-dominantly inherited maturity onset diabetes of the young (MODY-8) with exocrine dysfunctions. These single base deletions lead to a premature stop codon, resulting in truncated proteins [20, 21]. Affected family members typically develop diabetes, characterized by primary β-cell dysfunction, although transgenic mice over-expressing truncated BSDL failed to develop a MODY-8 phenotype [22]. This means that the pathogenic mechanisms involved are more complex than a simple loss of BSDL function. Truncated BSDL showed a high propensity to form aggregates both intra- and extra-cellularly [23, 24]. Therefore MODY-8 pancreatic syndrome may be caused by BSDL misfolding with a negative gain-of-function effect of truncated protein in the pancreas. Further work using the HEK293-T cell line showed that following secretion, the truncated BSDL was re-internalized and degraded in lysosomes [24]. Such internalization may reduce the viability of both acinar and endocrine pancreatic cells. Another study [25] found that a recombined allele of the *BSDL* gene and its pseudogene BSDLP conferred susceptibility to chronic pancreatitis. The resulting BSDL hybrid showed impaired secretion, prominent intracellular accumulation and induced autophagy.

The presence of short forms of BSDL in human pancreatic juice has been reported by Meyer [26], and Duang and Borgström [27]. Furthermore in 2000, Pasqualini et al. showed that MiaPaCa-2 cells expressed an immunoreactive form of BSDL of approximately 70 kDa, which corresponds to the 1.8 kb cDNA obtained by RT-PCR using a pair of primers covering the full-length mRNA encoding the human BSDL [28]. However, the sequence of the transcript of 1.8 kb obtained from MiaPaCa-2 cells was shown to differ from that of the human pancreatic BSDL cDNA (2.2 kb) within the 3′-end region, which encodes mucin-like VNTR sequences [5]. The amino-acid sequence deduced from this 1.8 kb RT-PCR product was homologous with that of BSDL, except for a deletion of 110 amino acids occurring within VNTR. A recent publication demonstrated that characterization of mutations within a GC-rich domain of the genome requires a careful examination of electropherograms [29]. In light of this, we hypothesized that the 1.8 kb transcript observed more than a decade ago in pancreatic tumor cells, might result from a modification of the BSDL gene sequence, overlooked due to the GC-richness within the VNTR. Therefore we attempted to characterize sequence modifications in the C-terminal domain of BSDL occuring in pancreatic pathologies, in part pancreatic ductal adenocarcinoma (PDAC).

## RESULTS

### Detection of BSDL transcripts in human pancreatic tumor cells

**Figure 1:**
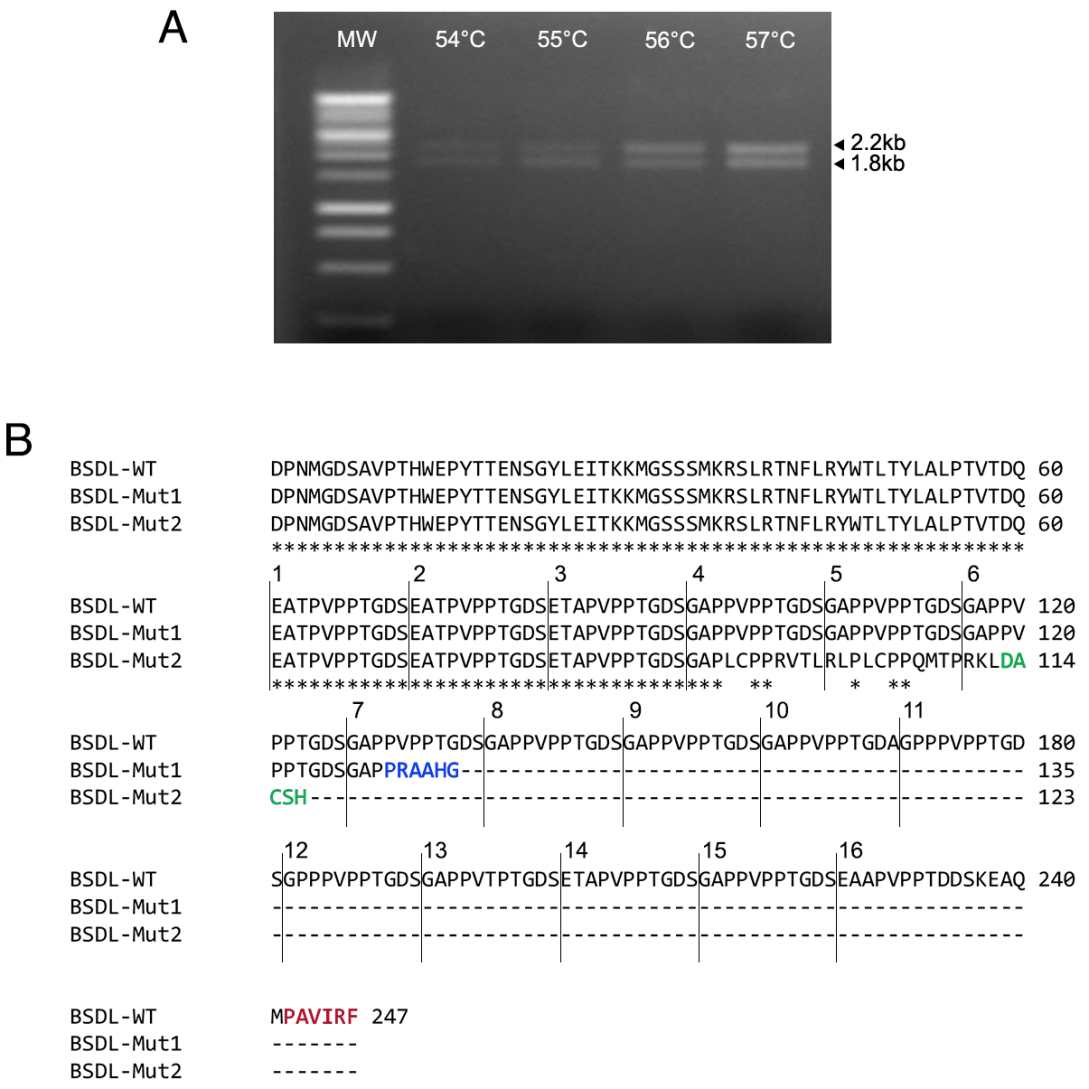
Genetic alterations within the DNA sequence of BSDL **A.** Sequences encoding BSDL were amplified from the RNA extracted from pancreatic tumor SOJ-6 cells using RT-PCR and specific primers. The PCR was performed in a personal Robocycler Gradient 96 (Stratagene). Following PCR, migration on agarose gel was performed to visualize amplified fragments. **B.** Amplicons were purified and their amino-acid sequences deduced after Sanger sequencing. The BSDL-WT amino-acid sequence corresponds to the 2.2 kb amplicon associated with the BSDL-Mut1 sequence, and the BSDL-Mut2 to that of the 1.8 kb band. Star symbol indicates the identical amino-acids. Only the C-terminal sequences are shown.

To characterize BSDL transcripts in PDAC, we used specific primers of the full length BSDL cDNA to perform RT-PCR on RNA extracted from the SOJ-6 pancreatic cell line, in which BSDL secretion is partly impaired [30]. Three amplicons were obtained and Sanger sequenced. The first transcript, at approximately 2.2 kb, corresponded to the published sequence of the full length BSDL cDNA. An associated second transcript displayed an insertion of a cytosine residue at position 1885 in repeat 7 (BSDL-Mut1) leading to a premature stop codon and a different C-terminal sequence *i.e*. PRAAHG (Pro-Arg-Ala-Ala-His-Gly) (Figure 1). This differed from the PAVIRF (Pro-Ala-Val-Ile-Arg-Phe) found at the C-terminal end of the full-length wild-type BSDL (BSDL-WT), which represents the normal C-terminal amino-acid sequence of human BSDL [5]. The third transcript of 1.8 kb presented a cytosine residue deletion at nt1785. This mutation found in RNA extracted from SOJ-6 pancreatic tumor cells also gives rise to a premature stop codon and to a truncated protein with a different C-terminal sequence (Figure 1) *i.e*. DACSH (Asp-Ala-Cys-Ser-His) (BSDL-Mut2). This transcript possibly corresponds to the short sequence described by Pasqualini et al. [28], in MiaPaCa-2 pancreatic tumor cells. The C-terminal sequences of the three transcripts are given in Supplementary Figure S1.

### Detection of BSDL in human pancreatic tumor tissues

Data obtained using human pancreatic tumor MiaPaCa-2 and SOJ-6 cell lines may not reflect physio-pathological situations. However the BSDL-Mut2 variant had not been detected to date in patients therefore we focused on BSDL-Mut1 expression. Consequently we collected pancreatic tissue samples from a cohort of 49 patients; 32 patients with resected PDAC, 6 were patients with non-PDAC pancreatic cancers (ampulloma, malignant endocrine carcinoma, mucinous cystadenoma), and 11 patients with non-malignant pancreatic diseases (non-MPD, intraductal papillary mucinous tumor of the pancreas [IPMT] or chronic calcifying pancreatitis [CCP]). Touch-down (TD)-PCR then allowed the amplification of DNA extracted from these tissues, using two pairs of primers, targeting the full exon 11 from 5′-end to 3′-end and the overlapping region between intron 10/exon 11 and 3′-end of exon 11 (Table 1). Sequencing of the resulting amplicons indicated that six out of 32 patients (18.8%) with PDAC (Supplementary Table S1) and 1 patient over 11 with non-MPD (9.1 %, Supplementary Table S2) showed an insertion of a cytosine residue in repeat 4, or in repeat 5, or in repeat 8, or in repeat 9. Whatever the position of the cytosine residue insertion, this generated a premature stop codon leading to a truncated protein with identical C-terminal sequence (*i.e* PRAAHG). This sequence correlated with that of BSDL-Mut1 above characterized in SOJ-6 cells, and will now be referred to as ‘BSDL with an apparent cytosine insertion’ or BSDL-ApInsC. Note that whenever possible mRNA was extracted from pancreatic tumor tissue samples and retrotranscripted cDNA thus obtained displayed identical sequence than that of the corresponding DNA.

**Table 1:** Primers used for amplification and Sanger sequencing of exon 11 BSDL gene

*Forward primers*	
gDNA:	5′ TCTTCTCACTCTGCAGGGACC 3′
cDNA:	5′ GAACCAACTTCCTGCGCTAC 3′
*Reverse primer*	
gDNA/cDNA:	5′ CAGGGGTATGAGGCTTTATTCA 3′

Examining a cohort of Norwegian individuals, Raeder et al. [21] detected a germline insertion of a base in VNTR of BSDL with an allelic frequency of some 7 %. Therefore we also examined a cohort of French people constituted of 92 individuals of whom 44 were patients suffering with infertility, 40 were patients with glioblastoma and 8 were patients with chronic calcifying pancreatitis (CCP) (see [31] for clinical data on these populations and Supplementary Table S2). DNA extracted from the whole blood of this cohort showed a wild-type amplification of BSDL DNA.

Taken as a whole such data suggest that the C insertion in VNTR of BSDL detected in tumor DNA and not in blood DNA, albeit different cohorts were examined, could be a somatic mutation.

### Development of specific antibodies against the C-terminal sequences of the BSDL-WT and the BSDL-ApInsC

**Figure 2:**
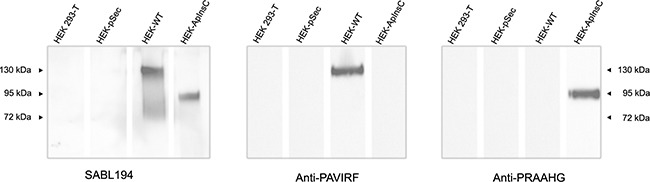
Transfected HEK293-T cell supernatants reactivity with SAB L194, anti-PAVIRF and anti-PRAAHG antibodies HEK293-T cells were transfected with empty pSecTag vector (HEK-pSec), with cDNA coding for the BSDL-WT (HEK-WT) or with cDNA encoding the BSDL-ApInsC (HEK-ApInsC) inserted in the pSec-Tag vector. Cells were cultured to confluence and supernatants were removed. Conditioned medium (30 μg protein per well lane) was loaded for electrophoresis on SDS-PAGE gels and transferred onto a nitrocellulose membrane. After saturation, the membrane was incubated overnight with the primary antibody as indicated, then washed and incubated with the required secondary POD antibody before detection.

**Figure 3:**
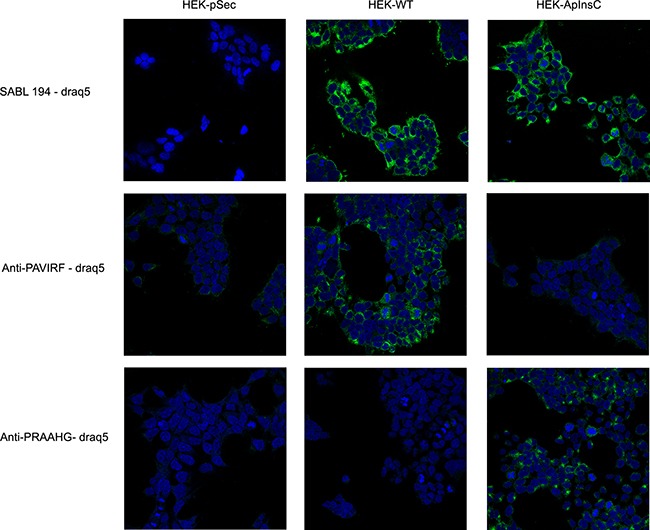
Transfected HEK293-T cells reactivity with SAB L194, anti-PAVIRF and anti-PRAAHG antibodies HEK-pSec, HEK-WT and HEK-ApInsC cells were seeded on 1.4 cm-diameter cover slips in 12-well plates. 48 hours later, cells were washed with PBS and then fixed and saturated with PBS 4% BSA. Cells were then incubated with primary antibodies diluted with PBS 1% BSA, and bound antibodies were detected using a secondary antibody to rabbit IgG coupled to Alexa 488. Nuclei were labeled with Draq5 (1 μM, 10 min, 37°C). Examination was performed using a Leica SP5 confocal microscope.

**Figure 4:**
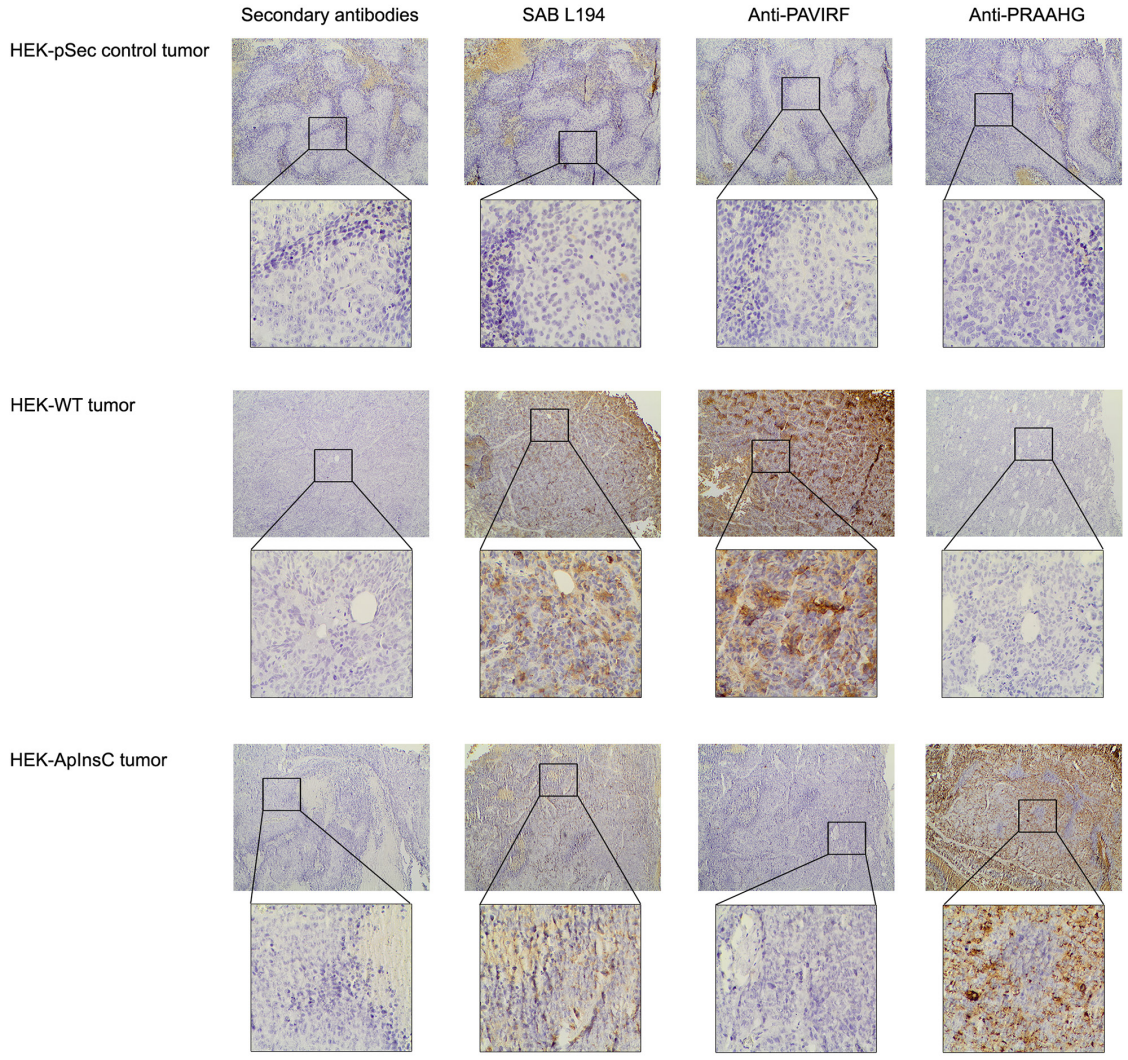
Immunohistochemistry on tumors xenotransplanted in nude mice HEK-pSec, HEK-WT and HEK-ApInsC cells were xenotransplanted in nude mice to obtain HEK-pSec, HEK-WT and HEK-ApInsC tumors respectively. Tumors were excised at 500 mm^3^, then formalin-fixed and paraffin embedded. All immunohistochemistry steps were performed on 5μm sections on the Leica BOND III automated system according to the manufacturers' instructions. Anti-PAVIRF and anti-PRAAHG antibodies were used at 0.2μg/ml and SAB L194 antibody at 1/5000 in Leica diluents. Nuclei were counterstained with Mayer's hematoxylin.

Because of the differences between the C-terminal sequences of BSDL-ApInsC and of BSDL-WT, we generated polyclonal antibodies directed against the whole BSDL, against the C-terminal sequence of the BSDL-WT, and against that deduced from the nucleotide sequence of the Mut1 variant (BSDL-ApInsC). These polyclonal antibodies are referred to as SAB L194, anti-PAVIRF and anti-PRAAHG, respectively, and were reactive both with SOJ-6 cells and with tumors obtained after xenotransplantation of SOJ-6 cells in nude mice (data not shown). For recombinant protein expression, we cloned and inserted the cDNA of BSDL-WT and BSDL-ApInsC within the pSecTag2-vector for transfection into HEK293-T cells. These transfected cells expressed recombinant full-length BSDL (BSDL-WT) and mutated BSDL (BSDL-ApInsC) in cell supernatant, both of which were recognized by SAB L194 polyclonal antibody to human BSDL (Figure 2). Recombinant BSDL-WT and BSDL-ApInsC displayed identical specific activity on a synthetic substrate and did not significantly affect HEK293-T cell growth (data not shown). However antibodies to PAVIRF recognized only BSDL-WT (Mr approx 120-130 kDa) and antibodies to PRAAHG bound only to BSDL-ApInsC (Mr approx 80-90 kDa, Figure 2). Immunocytochemistry also showed that HEK293-T cells expressing BSDL-WT (HEK-WT) were reactive to anti-PAVIRF antibodies whereas HEK293-T cells expressing BSDL-ApInsC (HEK-ApInsC) were recognized by anti-PRAAHG antibodies (Figure 3). Subsequently, and to evaluate the usefulness of these antibodies in immunohistochemistry, we xenotransplanted HEK293-T cells into nude mice and resected the tumor when palpable. Control tumors (*i.e*. tumors obtained after transplantation of HEK293-T cells transfected with the empty pSecTag2 vector) were not reactive to SAB L194, anti-PAVIRF or anti-PRAAHG antibodies. However, tumors recovered from mice xenotransplanted with HEK-WT and HEK-ApInsC cells were both reactive to SAB L194 and also to anti-PAVIRF or anti-PRAAHG antibodies respectively (Figure 4). Taken together these data confirm the specificity of antibodies and their usefulness in both western-blotting and immunohistochemistry experiments.

### Detection of the BSDL-WT and the BSDL-ApInsC in human pancreatic tumor tissues

**Figure 5:**
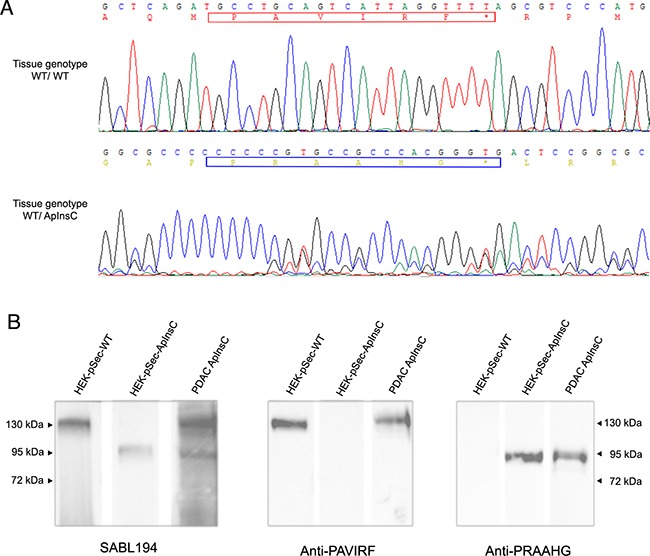
Immunoreactivity of human pancreatic tissue homogenates **A.** Analysis of electropherograms obtained after the sequencing of amplicons (obtained from tumoral genomic DNA) by the Sanger technique on samples from the studied patients with wild-type alleles (upper panel) or with wild-type / ApInsC alleles (lower panel). The electropherogram displays only the area where the apparent cytosine insertion occurs. **B.** Pancreatic tumor tissue from a patient (WT/ApInsC tissue genotyped) was homogenized before SDS-PAGE analysis (50 μg loaded on to the gel) and immunodetection with antibodies was as specified. The band migration was compared to that obtained with transfected HEK293-T cells (see Figure 2). The detection was performed with the required secondary POD antibody.

**Figure 6:**
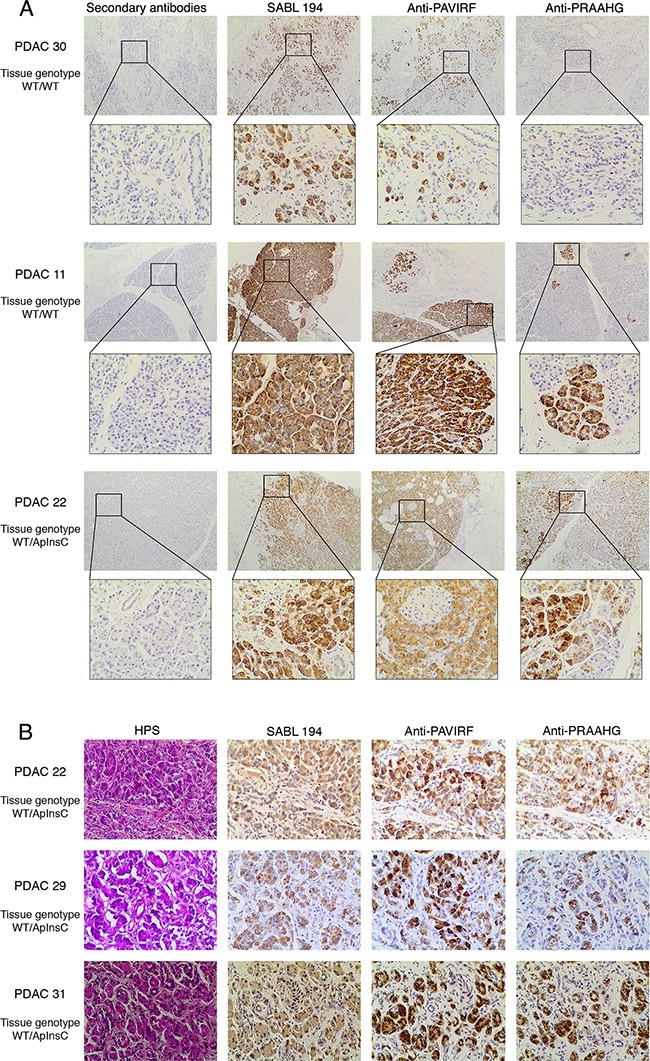
Immunohistochemistry on human pancreatic tumors **A**. Formalin-fixed, paraffin-embedded tissue sections of pancreatic tissue from genotyped WT/WT or WT/ApInsC tissue patients (PDAC 11, 22 and 30 refer to Supplementary Table S1) were stained by anti-PAVIRF, anti-PRAAHG and SAB L194 antibodies. All immunohistochemistry steps were performed on the Leica BOND III automated system according to the manufacturers' instructions. Anti-PAVIRF and anti-PRAAHG antibodies were used at 0.2 μg/ml and SAB L194 antibody at 1/1500 in Leica diluent. **B**. Formalin-fixed, paraffin-embedded tissue sections of dysplastic pancreatic tissue from genotyped WT/ApInsC tissue patients (PDAC 22, 29 and 31, refer to Supplementary Table S1) were stained by anti-PAVIRF, anti-PRAAHG and SAB L194 antibodies. All immunohistochemistry steps were performed as above.

We continued our investigations using human pancreatic tumor tissues. Proteins extracted from tumor tissue of a patient with tissue DNA presenting with both the wild-type allele (BSDL-WT) and the allele with a cytosine residue insertion in repeat 9 (BSDL-ApInsC) (Figure 5A) were submitted to SDS-PAGE and western blotting. As shown on Figure 5B two protein bands at 130 kDa and 90 kDa were reactive to SAB L194 (Figure 5B, left). However only the slow migrating band was reactive with anti-PAVIRF (middle) whereas the lower Mr band only reacts with anti-PRAAHG (Figure 5B, right). We consequently examined tissue sections from 18 patients with PDAC for reactivity to SAB L194, anti-PAVIRF and anti-PRAAHG antibodies. Tissue sections from 5 out of 18 patients (27.8%) were WT/WT genotyped (Figure 6A, patient PDAC30, see Supplementary Table S1) and also showed reactivity to SAB L194 and to anti-PAVIRF antibodies but not to anti-PRAAHG antibodies. Tissue sections from 5/18 (27.8%) patients were genotyped WT/ApInsC. These sections were all reactive to SAB L194 and to anti-PAVIRF. Overall they were strongly reactive to anti-PRAAHG antibodies (Figure 6A, patient PDAC22, see Supplementary Table S1). Interestingly, tissue sections from the remaining 8/18 (44.4%) patients were WT/WT genotyped and reactive to SAB L194 and to anti-PAVIRF antibodies but also displayed sporadic areas (some cells or small areas) that were reactive to anti-PRAAHG antibodies (Figure 6A, patient PDAC11, see Supplementary Table S1). These cells or areas, positive to anti-PRAAHG antibodies, presented a morphological acinar aspect that was not yet neoplastic but showed a lost of nuclear polarity, suggesting that truncated BSDL may be expressed early during pancreatic carcinogenesis. As expected anti-PRAAHG along with anti-SABL194 and anti-PAVIRF reactivity can be detected in dysplastic areas of the tumor from patients expressing BSDL-ApInC (Figure 6B, patient PDAC22, 29 and 31). Nevertheless we never saw any reactivity of anti-PRAAHG or anti-PAVIRF antibodies with neoplastic cells with ductal differentiation (Supplementary Figure S2). Taken as a whole, 13/18 patients with PDAC (72.2%) were positive to SAB L194, anti-PAVIRF and to anti-PRAAHG antibodies meaning that the truncated isoform of BSDL (BSDL-ApInsC), along with the normal form of the enzyme, is expressed in these tissues or at least by some cells within the tumor mass. Genotyped WT/WT tissues from four patients were neither reactive to SAB L194, nor to anti-PAVIRF and to anti-PRAAHG antibodies and tissue examination showed that these samples were mainly stromal (reactive to anti-α-SMA antibodies) with an absence of acinar tissue. Tissues from two patients with pancreatic neuro-endocrine tumors were not reactive to SAB L194, anti-PAVIRF, or to anti-PRAAHG antibodies (Supplementary Figure S3).

**Figure 7:**
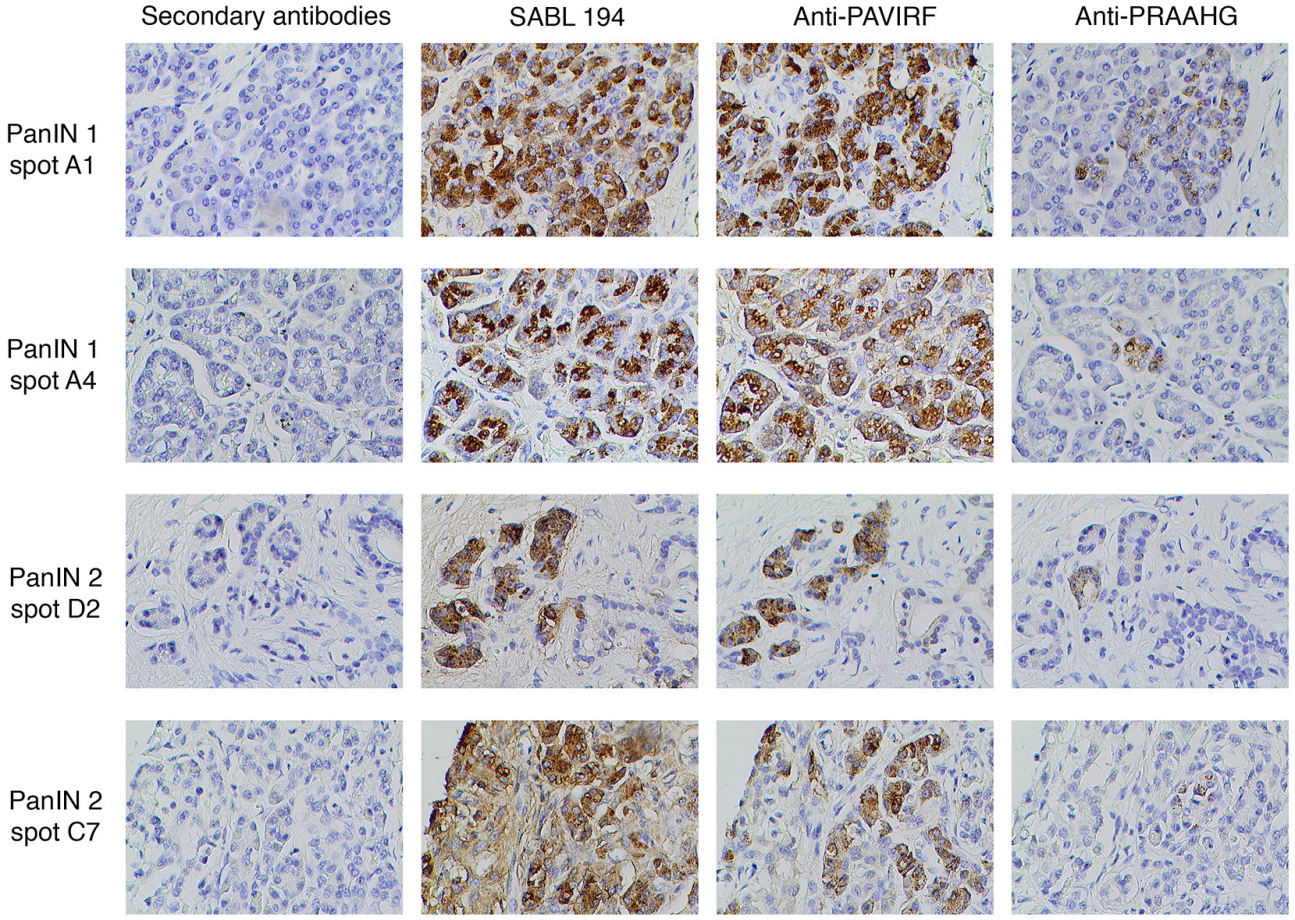
Immunohistochemistry on human Pancreatic Intraductal Neoplasia (PanIN) tissues A commercial Tissues Micro Array of human PanIN tissues has been stained by anti-PRAAHG antibodies. All immunohistochemistry steps were performed as described in Figure 6. Spot number refers to spot position in the TMA.

To demonstrate the early expression of the truncated BSDL with the PRAAHG C-terminal sequence in pancreatic cancer we examined a commercial Tissue Micro-Array (USBiomax) with Pancreatic Intraductal Neoplasia (PanIN), 10 spots were human tissue with stage 1 PanIN (PanIN-1, very early stage) and 7 were stage 2 (PanIN-2). Staging of PanIN tissues were confirmed by a senior anatomo-pathologist co-authoring this manuscript (JDG). 7/10 PanIN-1 (70 %) and 5/7 PanIN-2 (71.4 %) were reactive to anti-PRAAHG antibodies (Figure 7). Noteworthy normal areas in PanIN tissues were not reactive to anti-PRAAHG antibodies. We also examine blood DNA, tissue DNA and anti-PRAAHG reactivity on 4 patients with PDAC. DNA extracted from the blood cells and the whole blood of 3 of these patients showed a wild-type amplification of the BSDL gene associated with an InsC in DNA extracted from tumor tissue and/or with a reactivity to anti-PRAAHG antibodies in immunohistochemistry experiments. These data strongly suggest that the cytosine insertion in VNTR of BSDL is a somatic event appearing in early stage of the pancreatic cell transformation. A somatic event also explain the fact that the InsC cannot be detected 1/ in the genetic material extracted from blood samples of controls and patients afflicted with a glioblastoma or with IPMT/CCP and 2/ in normal area of PanIN tissues. However the last patient showed an InsC in blood cells and whole blood DNA with a reactivity to anti-PRAAHG antibodies. This means that some individual may inherit the InsC in VNTR of *BSDL*.

### KRAS status of the PDAC cohort

Because the InsC as a somatic event may be due to a genetic instability we also examine the KRAS status of 19 patients with a PDAC. For this purpose pancreatic tumoral tissue has been microdissected to only analyze the tumor cells for KRAS mutation on exon 2, 3 or 4 [31]. 74% of examined tissues were mutated on KRAS. As shown on Supplementary Table S1, 5/19 (26.3 %) samples mutated on KRAS were also reactive to anti-PRAAHG antibodies. However 3/19 (15.8 %) WT for KRAS showed a strong reactivity to anti-PRAAHG antibodies and also displayed insC in whatever VNTR of BSDL, 3/19 samples (15.8 %) had mutated KRAS and no reactivity to anti-PRAAHG antibodies. These data showing no relationship between KRAS mutations and InsC suggested that the latter event may not be due to a genetic instability.

### Detection of the BSDL-WT and the BSDL-ApInsC in human pancreatic juice

**Figure 8:**
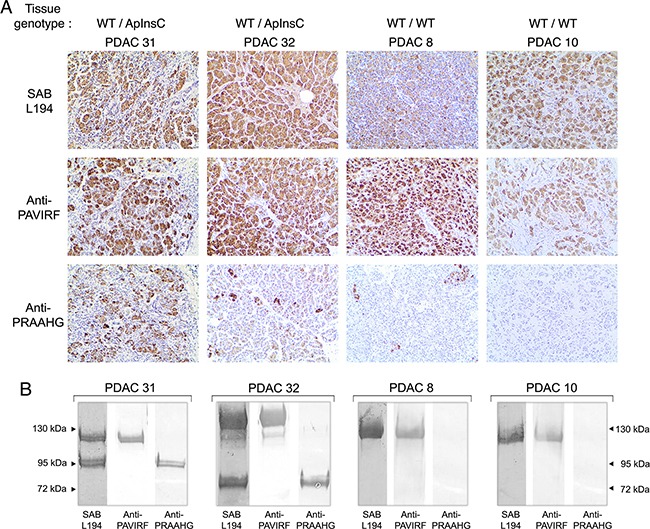
Immunoreactivity of human tissues and pancreatic juices from the same patients **A.** Formalin-fixed, paraffin-embedded tissue sections of pancreatic tissue from genotyped WT/ApInsC (PDAC 31 and 32) or WT/WT (PDAC 8 and 10) tissue patients were stained by anti-PAVIRF, anti-PRAAHG and SAB L194 antibodies. All immunohistochemistry steps were performed as described in Figure 6 legend. **B.** Human pancreatic juice collected from patients PDAC 31 and 32 (WT/ApInsC tissue genotyped) and from patients PDAC 8 and 10 (WT/WT tissue genotyped) was analyzed on SDS-PAGE (approx. 50 μg protein loaded). Immunodetection was performed via incubation first with primary antibodies as specified then with the required secondary alkaline phosphatase antibodies.

Deletion of the C-terminal end of BSDL significantly decreased the expression of the protein [32]. SOJ-6 cells, from which BSDL-ApInsC (BSDL-Mut1) was detected, also poorly secreted the enzyme. Although this poor secretion can be attributed to a defect in vesicular transport of secretory proteins [30], it is tempting to speculate that the BSDL-ApInsC isoform may not be secreted by acinar cells. To check this specific point, pancreatic juice samples were collected during surgery and analyzed by western blot using antibodies generated in our laboratory (Figure 8). Four patients with PDAC were examined; two with the WT/ApInsC tissue genotype and with pancreatic tumor tissue reactive to the anti-PRAAHG (patients PDAC31 and 32, Figure 8A, see Supplementary Table S1) and two with the WT/WT tissue genotype and with pancreatic tumor tissue not reactive or poorly reactive to the anti-PRAAHG (PDAC8 and 10, Figure 8A, see Supplementary Table S1). Samples from patients PDAC31 and 32, displayed two bands around 120-130 and 75-95 kDa, both reactive with SAB L194 antibodies (Figure 8B). Interestingly the lower band was reactive with anti-PRAAHG antibodies (insertion of a cytosine residue in repeat 9 for the patient PDAC31; insertion of a cytosine residue in repeat 5 for the patient PDAC32) whereas the upper band was only recognized by anti-PAVIRF antibodies. In WT/WT genotyped patients PDAC8 and 10, in our experimental conditions, only the higher band could be detected, associated with reactivity to SAB L194 and anti-PAVIRF antibodies (Figure 8B). These data demonstrate that antibodies to PRAAHG peptide sequence were only reactive with BSDL-ApInsC, and that this short isoform of BSDL is secreted in pancreatic juice and appears to be specifically expressed in PDAC.

## DISCUSSION

With more than 277,000 new cases per year worldwide, pancreatic cancers represent 10% of all digestive cancers; 90% of these are PDAC (www.cancer.org). The survival rate is dramatically low with a case-fatality ratio of about 0.9. PDAC could be the second cause of death by cancer by the year 2030 [33] and today has a 5-year survival rate of less than 4% in western countries [34]. Its poor prognosis is mainly due to a lack of response to currently available therapies [35, 36] and to a very low curative resection rate (15% of patients) due to nonspecific symptoms, a lack of early biological markers, delayed diagnosis and metastasis formation. Patients diagnosed with advanced or metastatic disease are not eligible for surgery (85% of patients) and show median survival rates ranging from 6 to 9 months [37, 38]. For those patients with inoperable cancer, the main treatment remains standard chemotherapy. Pancreatic adenocarcinomas, as well as precancerous lesions, occur as a result of alterations affecting genes necessary to maintain cellular functions. These changes can include mutations, deletions or epigenetic modifications, and result in a gain or loss of function of genes. Alterations can be inherited (familial cancers, predispositions) or acquired, but in either case each stage of disease is associated with one or more type of alteration. The evolution and accumulation of these alterations explains the increasing invasiveness of lesions. In 80-90% of pancreatic adenocarcinomas the KRAS gene on chromosome 12 is mutated [39]. The most common mutations seen in pancreatic cancer are primarily those affecting codon 12, followed by mutations to codons 13 and 61 [40]. Found in precancerous lesions, these mutations are responsible for constitutive activation of KRAS signaling and contribute to tumor development [41, 42].

Here we showed that an apparent insertion of a cytosine residue in the BSDL (or CEL) gene seems associated with PDAC, in part with pre-neoplastic lesions of the pancreas. This apparent insertion was detected upon careful examination of the GC-rich sequence characterizing VNTR in the BSDL gene. This mutation can be located in any of the VNTR; in the present study insertions into VNTR 4, 5, 8 or 9 were found. Any position of this apparent insertion leads to a reading frame shift generating a premature stop codon. It is not yet known whether this apparent insertion results from a true insertion of a cytosine residue or from the deletion of some repeated sequences leaving a cytosine residue in place. However this apparent cytosine insertion generates a new C-terminal sequence of the resulting truncated BSDL. Such a PRAAHG sequence in BSDL has already been characterized as a gene polymorphism of the milk counterpart of the pancreatic BSDL, but was not associated with any breast pathology [43]. Furthermore a single-base insertion with an allelic frequency of about 7% generating a premature stop codon with a frame shift leading to a truncated protein has also been observed in a Norwegian population [21]. This germline insertion had not been associated with a specific pancreatic pathology albeit a single-base deletion in VNTR 1 or 4 was associated with a diabetic and pancreatic exocrine dysfunction (referred to as MODY-8) in two families [21]. Diabetic patients with a base insertion in repeat BSDL exon 11 VNTR displayed predisposition to exocrine dysfunctions [21]. In the present study we showed that this germline C insertion albeit rare (the InsC has been observed in the whole blood of one people over the 110 examined: *i.e*. <1 %) may be observed in our French cohorts. For now we cannot conclude that this germline C-insertion may predispose to PDAC. Nevertheless we demonstrated that this insertion giving rise to a truncated BSDL with PRAAHG C-terminal motif also occurs within 72.2 % of patients with PDAC that were examined. For PDAC affected French patients this insertion is likely not representative of a germline mutation. One hypothesis to explain the nature germinal or somatic of the insertion could be a genetic variation between examined populations, one coming from northern Europe and the second from southern Europe. This last point may be illustrated by many studies in part those of Tian *et al*. [44] and Seldin *et al.* [45]. How does a germline insertion in *BSDL* VNTR may predispose to an exocrine pancreatic dysfunction in diabetic populations of northern Europe is still an open question [22–24]. The outcome of these patients is unknown and would have deserved special attention. What could be the pathophysiological implication of the same insertion, but now mainly somatic, and occuring later on during early stage of pancreatic carcinogenesis is an other question.

We recognized that this occurrence may not reflect pathophysiological conditions; this insertion may not be detected in PDAC tissue DNA due to either a low number of tumor cells expressing truncated BSDL, or to the presence of stromal cells that do not express BSDL, both diluting the signal. Therefore we hypothesized that determining the tissue expression of truncated BSDL may be more informative of the occurrence of this insertion. For this purpose we designed two peptides, one with the normal C-terminal sequence of BSDL and one with a C-terminal sequence common to truncated BSDL whatever the position of the apparent cytosine insertion. Antibodies were made to both these sequences and specifically recognized BSDL expressed by HEK293-T cells once transfected with vectors encoding either BSDL-WT (recognized by anti-PAVIRF antibodies) or BSDL-ApInsC (recognized by anti-PRAAHG antibodies). With these highly specific tools we examined human pancreatic tumoral tissues. Immunohistochemistry experiments demonstrated that anti-PAVIRF antibodies bound to tissues of WT/ApInsC or WT/WT genotyped individuals whereas anti-PRAAHG antibodies only recognize tissues of WT/ApInsC patients. Interestingly anti-PRAAHG antibodies also recognized some tissue areas or cells of WT/WT genotyped patients with PDAC, suggesting that anti-PRAAHG antibodies are more appropriate to detect C-terminal sequence modification in BSDL than is the PCR/sequencing to detect the cytosine apparent insertion in *BSDL* VNTR. Indeed some 72% of tissues coming from patients with PDAC were reactive to anti-PRAAHG antibodies. Examination of the areas of tissue positive to anti-PRAAHG antibodies showed a still normal acinar morphology without neoplasticity, albeit cell nuclei have lost polarity. It could be that the apparent cytosine insertion in VNTR of the BSDL gene occurs early during carcinogenesis. This point is strengthened by the reactivity of dysplastic area in pancreatic tumor tissue and in PanIN-1 and PanIN-2 to antibodies against PRAAHG C-terminal sequence. However neoplastic cells with ductal differentiation do not react, in our experimental conditions with anti-PRAAHG and anti-PAVIRF antibodies. One hypothesis to explain this point is that the expression of truncated BSDL (and more generally BSDL as suggested by the lack of anti-PAVIRF reactivity) is repressed enough to become undetectable by our antibodies against peptides, following the ductal differentiation of acinar tumor cell. Nevertheless the expression of BSDL in these cells is still sufficient to be detected by antibodies mainly directed against sugar harbored by VNTR, *i.e.* mAb16D10 or pAbL64 [46, 47]. Consequently the expression of truncated BSDL with PRAAHG C-terminal sequence may be a marker of pre-neoplastic states occurring in pancreatic carcinogenesis. In the light of deleterious effects of truncated/mutated BSDL in the pancreas, the retention or recapture of which may cause diseases such as MODY-8 diabetes (due to inherited deletion in VNTR) [20, 21, 23, 24] or chronic pancreatitis (due to a recombined allele of the BSDL gene and the BSDL pseudogene) [25], we examined the presence of the shorter isoform of BSDL in pancreatic juices from patients with PDAC. A response to the anti-PRAAHG antibodies was detected in pancreatic juices collected from WT/ApInsC genotyped patients and is associated with the truncated isoform of BSDL. This suggests, therefore, that the expression of the PRAAHG C-terminal sequence does not impact on BSDL protein expression and secretion, and may not affect cell behavior. However the exact role of this truncated isoform of BSDL in PDAC development requires further examination. Albeit difficult, the detection of the PRAAHG-reactive isoform of BSDL either by ELISA in pancreatic juice obtained after endoscopic examination or immuno-histochemistry on pancreatic biopsies may be a new tool in PDAC diagnosis.

## MATERIALS AND METHODS

### Cell lines

SOJ-6 cells originating from human pancreatic adenocarcinoma were grown in RPMI 1640 medium supplemented with 10% fetal calf serum (FCS), penicillin (100 U/ml) and streptomycin (100 μg/ml). Human embryonic kidney (HEK) cells expressing the SV40 large T antigen (HEK293-T) (ATCC CRL-11268) were maintained in DMEM medium supplemented with 10% FCS, glutamine (2 mM), penicillin (100 U/ml) and streptomycin (100 μg/ml). Pancreatic cancer TMA (catalog ref. BIC 14011a) came from USBiomax (Rockville, MD USA).

### Antibodies

Patented anti-PRAAHG antibodies were generated in rabbit using the nine carboxy-terminal amino acids of BSDL-ApInsC (GAPPRAAHG). Anti-PAVIRF antibodies were generated in rabbit using the twelve carboxy-terminal amino acids of BSDL-WT (CKEAQMPAVIRF). Polyclonal rabbit antiserum SAB L194 detects all BSDL isoforms (Eurogentec, Angers, France). Alkaline phosphatase-labeled goat antibodies to rabbit IgG, peroxidase (POD)-labeled goat antibodies to rabbit IgG and AlexaFluor488-labeled donkey antibodies to rabbit IgG were purchased from LifeTechnologies (St Aubin, France). DRAQ5™, a far-red fluorescent DNA dye was from Biostatus Limited (Shepshed, UK). Antibodies to α-SMA (clone 1A4) came from DakoCytomation A/S (Glostrup, Denmark).

### Control cohort

The French control group comprised blood samples from 44 individuals presenting with no cancer-related diseases. Subjects afflicted with infertility were aged between 16 and 54 years (mean = 30.9 years, SD = 8.3) and the male/female ratio was 13/1. Control samples were collected by the Department of Medical Genetics (Timone Hospital, Marseille) between January 2000 and October 2009.

### Pancreatic cancer samples

Tumor tissues samples were obtained after pancreatic resection (duodeno-pancreatectomy) from 32 patients diagnosed with pancreatic adenocarcinoma (Gastroenterology and Digestive Surgery departments, Timone Hospital, Marseille) between February 2007 and February 2014 (Supplementary Table S1). Patients were aged between 50 and 87 years (mean = 66.1 years, SD = 9.4) and the male/female ratio was 2/3. A definitive diagnosis of pancreatic adenocarcinoma was made after histochemical analysis of the resected tumor tissues. The average tumor size was 3.25 cm (SD = 1.2). WHO and TNM stages were determined by a senior pathologist with expertise in the field. One patient had a stage 1B localized tumor, 10 a stage 2A localized tumor, 16 stage 2B lymph node metastases, 3 were diagnosed at stage 4 (metastatic stage) and 2 were NC (data not communicated). Survival ranged from 3 to 72 months. According to the 2014 hospital records, 22 out of 32 patients with pancreatic cancer died and 10 were still alive at the time of the study. Whenever possible, pancreatic juice was collected during surgery and immediately frozen in liquid nitrogen.

### Non-malignant pancreatic disease samples

Patients displaying non-malignant pancreatic diseases with CCP or intraductal papillary mucinous tumor (IPMT) constituted the non-malignant pancreatic diseases (non-MPD) group. This cohort collected in the Gastroenterology department of Timone Hospital, Marseilles France, comprised 11 pancreatic tissues and 8 blood samples from patients aged between 40 and 76 years (mean age = 65.1 years, SD = 11.3) (Supplementary Table S2). The male/female ratio was 1.7/1. Four patients presented an IPMT, i.e. a cystic tumor of the pancreas with putative pre-neoplastic status, 14 had a CCP, and 1 presented a CCP complicated by a retention cyst. According to the hospital records, all these patients were still alive at the end of 2014.

### Non-pancreatic malignant disease samples

A cohort of 40 patients with non-pancreatic malignancies was examined. All 40 patients presented with glioblastoma. Blood samples were collected in the Neurobiology department of Timone Hospital. Patients (male/female ratio, 1/3) were aged between 20 and 81 years (mean = 58.9 years, SD = 12.5,) (see [31] for clinical data on these populations).

### Non-PDAC pancreatic cancers

The tissue samples were obtained after pancreatic resection (duodeno-pancreatectomy) in patients diagnosed with non-PDAC pancreatic cancers such as ampulloma, malignant endocrine carcinoma or mucinous cystadenoma. These patients constituted the non-PDAC pancreatic cancer (non-PDAC) group (n = 6). Subjects were aged between 60 and 83 years (mean = 72.2 years, SD = 8.7) and the male/female ratio was 1/1 (see [31] for clinical data on these populations). Control samples were collected in the Department of Gastroenterology and Digestive Surgery (Timone Hospital) between May 2009 and July 2013.

### Generalities on sampling

All tissue samples were from Caucasian subjects. Protocols for sample collection, sample anonymity, and genomic DNA analysis were approved by the local ethics committee. Written informed consent from all participants was obtained. Samples were stored in the CRO2 (Center for Research in Oncobiology and Oncopharmacology) biobank (agreement DC 2013-1857) or AP-HM (Assistance Publique - Hôpitaux de Marseille) BioBank (agreement AC-2013-1786). They were kept in a solution of RNA later ® (Life Technologies) from immediately after surgery and then subjected to snap-freezing in liquid nitrogen.

### Extraction of total RNA from SOJ-6 cell line

The extraction of total RNA was performed using the RNeasy Plus Mini kit (Qiagen) according to the protocol recommended by the supplier. Total RNA was quantified by optical density at 260 nm on LVisPlate Omega (BMG Labtech). The quality and integrity of extracted RNA was verified by agarose gel migration. Total RNA samples were then stored at −80°C until use.

### Extraction of genomic DNA (gDNA) from whole blood, blood cells and tissue samples

The gDNA was extracted from whole blood or frozen tissue using the QIAamp mini kit (Qiagen). The extraction was carried out using blood cells, 600 μl of whole blood or 25 mg of tissue as recommended by the supplier. Blood cells (Peripheral Blood Mononuclear Cells) were isolated from 5 ml peripheral blood. Red cells were removed and leukocytes were isolated by centrifugation (2000g, 15 min) after lysis of remaining red blood cells in isotonic buffer (10 mM Tris base, 5 mM MgCl2, 10 mM NaCl). The leukocyte pellet was washed and gDNA extraction was performed on dry pellet as above. The quantity and quality of extracted gDNA were determined by measuring the absorbance at 260 nm and 280 nm on LVisPlate Omega. gDNA samples were stored at −80°C until use.

### Amplification by RT-PCR

Reverse transcription was performed from 1 μg of total RNA in a reaction volume of 20 μl, containing 0.4 mM Oligo(dT)_15_ primer, 2 mM dNTP mix, 5 mM MgCl2, 20 U RNase inhibitor and 10 U AMV reverse transcriptase (AMV Reverse Transcriptase kit, Promega). The mixture was incubated 10 min at room temperature (RT), followed by incubations: one hour at 42°C, 5 min at 99°C and finally 10 min on ice. Then, the PCR was performed in a personal Robocycler Gradient 96 (Stratagene) in a reaction volume of 50 μl and comprised 2 μl of cDNA, 5 μl of 10X enzyme buffer, 10 μl of 5X GCmelt buffer (Ozyme), 200 nM of each primer, 0.4 mM of dNTP mix and 5 U Platinum Taq High fidelity DNA polymerase (Life Technologies). The program was applied as follows: 1 cycle of 5 min at 94°C; 35 cycles of 1 min at 94°C, 1 min at 54-57°C, 4 min at 68°C; 1 cycle of 10 min at 68°C. Following PCR, migration on agarose gel was performed to visualize the amplified fragment and check the size.

### Amplifications by TD-PCR

The TD-PCR was performed in a personal Mastercycler thermocycler (Eppendorf) in a reaction volume of 50 μl and comprised 100 ng gDNA or 2 μl of cDNA, 5 μl of 10X enzyme buffer, 10 μl of 5X GCmelt buffer (Ozyme), 200 nM of each primer, 0.4 mM of dNTP mix and 5 U Platinum Taq High fidelity DNA polymerase (Life Technologies). The program was applied as follows: 1 cycle of 5 min at 94°C; 9 cycles of 30 sec at 94°C, 30 sec at 64°C (−1°C per cycle), 1 min at 68°C; 30 cycles of 30 sec at 94°C, 30 sec at 55°C, 1 min at 68°C; and 1 cycle of 12 min at 68°C. Following PCR, migration on agarose gel was performed to visualize the amplified fragment and check the size.

### Amplicon purification and sequencing

The amplicons obtained were then purified using the DNA extraction kit (Millipore) after electrophoretic migration on agarose gel, according to the protocol recommended by the supplier. Sequencing of amplicons was carried out according to the Sanger method (Beckman Coulter Genomics, Meylan, France).

### KRAS Mutations

Kras mutations on exon 2, 3 and 4 of the KRAS gene were characterized as already described [31].

### Cell transfection

HEK293-T cells were transfected with pSecTag2B plasmid (Life Technologies) harboring BSDL-WT or BSDL-ApInsC using lipofectamine LTX (Life Technologies) as described by the manufacturer. Cells transfected with pSecTag2B without any insert were included in all experiments as a negative control. For stable BSDL expression, transfected HEK293-T cells were treated with 10 or 200 μg/ml Zeocin.

### Preparation of conditioned medium

HEK293-T cells were grown in DMEM medium supplemented with glutamine (2 mM), penicillin (100 U/ml), and streptomycin (100 μg/ml) to reach 60-70% confluence and then starved for 12 hours. Conditioned media were used for recombinant BSDL purification [48] and western blottings.

### Enzyme activity

Recombinant BSDL activity was recorded on a synthetic substrate as previously described [48].

### Immunofluorescence staining

Cells were seeded in appropriate medium on cover slips in 12-well plates (BD Falcon, Le Pont-de-Claix, France). Once adherent, cells were fixed (formaldehyde, 4% in PBS, at RT, 15 min) permeabilized with PBS-0.05% saponin (20 min at RT) and saturated (BSA, 4% in PBS, 30 min). The cells were then successively incubated with primary antibodies to PRAAHG or to PAVIRF peptides, or with SAB L194 antibody, for 120 min at RT and secondary AlexaFluor488-labeled antibodies to rabbit IgG for 60 min. The cell nuclei were labeled with 1 μM Draq5 for 30 min. Confocal microscopy acquisitions were performed using a Leica SP5 microscope coupled with a Leica scanning device (Leica Microsystems, Mannheim, Germany). Images were recorded with LAS AF Lite acquisition software and were analysed with the public-domain ImageJ software (NIH; http://rsb.info.nih.gov/nih-image/).

### Immunohistochemistry

Sections (5 μm) of formalin-fixed paraffin-embedded tissue were mounted on superfrost/plus slides from preheated water baths. All staining steps were performed on the Leica BOND III automated system (Leica Microsystems, Bannockburn, IL) according to the manufacturers' instructions. Anti-PAVIRF and anti-PRAAHG antibodies were used at 1/5000 in Leica diluents and SAB L194 antibody at 1/5000. Nuclei were counterstain with Mayer hematoxylin. The slides were dehydrated through 2 changes each of 70%, 95%, and 100% alcohol and 2 changes of xylene, before coverslipping.

### SDS-PAGE and western blottings

Proteins (30 μg of proteins from tissue homogenate or conditioned media or 5 μl of total pancreatic juice) in reducing SDS buffer were separated on 8% or 4-20% polyacrylamide gels (Thermo Scientific). After electrophoretic migration, proteins were transferred onto nitrocellulose membranes and processed for immunoblotting by using appropriate primary antibodies and alkaline-phophatase or POD-labeled secondary antibodies. After washes, membranes were developed with BCIP/NBT substrate or with a chemoluminescent substrate [49], visualized using a G:BOX-iChemi (SynGene) gel imaging device as described by the manufacturer, and analyzed with the NIH ImageJ program for band quantification.

## SUPPLEMENTARY FIGURES AND TABLES


