# Supplementary material for: Expression of truncated bile salt-dependent lipase variant in pancreatic pre-neoplastic lesions
Source: Oncotarget. 2016 Sep 1;8(1):536–51. doi: 10.18632/oncotarget.11777 (PMC5352176; doi:10.18632/oncotarget.11777)
Supplement: Supplementary file 1 [file oncotarget-08-536-s001.pdf]

Expression of truncated bile salt-dependent lipase variant in pancreatic pre-neoplastic lesions

SUPPLEMENTARY FIGURES AND TABLES

```
BSDL-WT         GGACCCCAACATGGGCGACTCGGCTGTGCCCACACACTGGGAACCCTACACTACGGAAAA 60
BSDL-Mut1       GGACCCCAACATGGGCGACTCGGCTGTGCCCACACACTGGGAACCCTACACTACGGAAAA 60
BSDL-Mut2       GGACCCCAACATGGGCGACTCGGCTGTGCCCACACACTGGGAACCCTACACTACGGAAAA 60
                ************************************************************

BSDL-WT         CAGCGGCTACCTGGAGATCACCAAGAAGATGGGCAGCAGCTCCATGAAGCGGAGCCTGAG 120
BSDL-Mut1       CAGCGGCTACCTGGAGATCACCAAGAAGATGGGCAGCAGCTCCATGAAGCGGAGCCTGAG 120
BSDL-Mut2       CAGCGGCTACCTGGAGATCACCAAGAAGATGGGCAGCAGCTCCATGAAGCGGAGCCTGAG 120
                ************************************************************
                                                       1
BSDL-WT         AACCAACTTCCTGCGCTACTGGACCCTCACCTATCTGGCGCTGCCCACAGTGACCGACCA 180
BSDL-Mut1       AACCAACTTCCTGCGCTACTGGACCCTCACCTATCTGGCGCTGCCCACAGTGACCGACCA 180
BSDL-Mut2       AACCAACTTCCTGCGCTACTGGACCCTCACCTATCTGGCGCTGCCCACAGTGACCGACCA 180
                ************************************************************
                                                  2
BSDL-WT         GGAGGCCACCCCTGTGCCCCCCACAGGGGACTCCGAGGCCACTCCCGTGCCCCCCACGGG 240
BSDL-Mut1       GGAGGCCACCCCTGTGCCCCCCACAGGGGACTCCGAGGCCACTCCCGTGCCCCCCACGGG 240
BSDL-Mut2       GGAGGCCACCCCTGTGCCCCCCACAGGGGACTCCGAGGCCACTCCCGTGCCCCCCACGGG 240
                ************************************************************
                       3                                4   ↓
BSDL-WT         TGACTCCGAGACCGCCCCCGTGCCGCCCACGGGTGACTCCGGGGCCCCCCCCGTGCCGCC 300
BSDL-Mut1       TGACTCCGAGACCGCCCCCGTGCCGCCCACGGGTGACTCCGGGGCCCCCCCCGTGCCGCC 300
BSDL-Mut2       TGACTCCGAGACCGCCCCCGTGCCGCCCACGGGTGACTCCGGGG---------------- 284
                ********************************************
                             5                                6
BSDL-WT         CACGGGTGACTCCGGGGCCCCCCCCGTGCCGCCCACGGGTGACTCCGGGGCCCCCCCCGT 360
BSDL-Mut1       CACGGGTGACTCCGGGGCCCCCCCCGTGCCGCCCACGGGTGACTCCGGGGCCCCCCCCGT 360
BSDL-Mut2       ------------------------------------------------------------

                                   7   ↓                             8
BSDL-WT         GCCGCCCACGGGTGACTCCGGGG-CCCCCCCCGTGCCGCCCACGGGTGACTCCGGGGCCC 419
BSDL-Mut1       GCCGCCCACGGGTGACTCCGGGGCCCCCCCCCGTGCCGCCCACGGGTGACTCCGGGGCCC 420
BSDL-Mut2       ------------------------------------------------------------

                                          9                                10
BSDL-WT         CCCCCGTGCCGCCCACGGGTGACTCCGGGGCCCCCCCCGTGCCGCCCACGGGTGACTCCG 479
BSDL-Mut1       CCCCCGTGCCGCCCACGGGTGACTCCGGGGCCCCCCCCGTGCCGCCCACGGGTGACTCCG 480
BSDL-Mut2       ------------------------------------------------------------

                                                11
BSDL-WT         GGGCCCCCCCCGTGCCGCCCACGGGTGACGCCGGGCCCCCCCCCGTGCCGCCCACGGGTG 539
BSDL-Mut1       GGGCCCCCCCCGTGCCGCCCACGGGTGACGCCGGGCCCCCCCCCGTGCCGCCCACGGGTG 540
BSDL-Mut2       ------------------------------------------------------------

                     12                               13
BSDL-WT         ACTCCGGGCCCCCCCCCGTGCCGCCCACGGGTGACTCCGGGGCCCCCCCCGTGACCCCCA 599
BSDL-Mut1       ACTCCGGGCCCCCCCCCGTGCCGCCCACGGGTGACTCCGGGGCCCCCCCCGTGACCCCCA 600
BSDL-Mut2       ------------------------------------------------------------

                           14                               15
BSDL-WT         CGGGTGACTCCGAGACCGCCCCCGTGCCGCCCACGGGTGACTCCGGGGCCCCCCCTGTGC 659
BSDL-Mut1       CGGGTGACTCCGAGACCGCCCCCGTGCCGCCCACGGGTGACTCCGGGGCCCCCCCTGTGC 660
BSDL-Mut2       -------------------------------------------------CCCCCCTGTGC 295
                                                                 ***********
                                 16
BSDL-WT         CCCCCACGGGTGACTCTGAGGCTGCCCCTGTGCCCCCCACAGATGACTCCAAGGAAGCTC 719
BSDL-Mut1       CCCCCACGGGTGACTCTGAGGCTGCCCCTGTGCCCCCCACAGATGACTCCAAGGAAGCTC 720
BSDL-Mut2       CCCCCACGGGTGACTCTGAGGCTGCCCCTGTGCCCCCCACAGATGACTCCAAGGAAGCTC 355
                ************************************************************

BSDL-WT         AGATGCCTGCAGTCATTAGGTTTTAGCGTCCCATGAGCCTTGGTATCAAGAGGC 773
BSDL-Mut1       AGATGCCTGCAGTCATTAGGTTTTAGCGTCCCATGAGCCTTGGTATCAAGAGGC 774
BSDL-Mut2       G-ATGCCTGCAGTCATTAA----------------------------------- 373
                . ****************.
```

Supplementary Figure S1: Nucleotide sequence of transcripts isolated from pancreatic tumor SOJ-6 cell line. The BSDL-WT sequence corresponds to the 2.2 kb amplicon associated with the BSDL-Mut1 sequence and the BSDL-Mut2 to that of the 1.8 kb band (see Figure 1). Number represents the repeated sequences in the VNTR of *BSDL*.

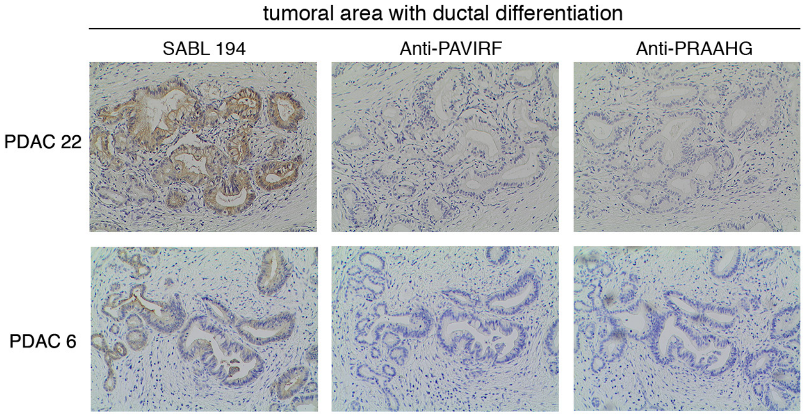

Supplementary Figure S2: Immunohistochemistry on pancreatic tumor area with ductal differentiation. Formalin-fixed, paraffin-embedded tissue sections of dysplastic pancreatic tissue from genotyped WT/ApInsC patients (PDAC 22 and 29, refer to Supplementary Table S1) were stained by anti-PAVIRF, anti-PRAAHG and SAB L194 antibodies. All immunohistochemistry steps were performed as described in Figure 6A legend.

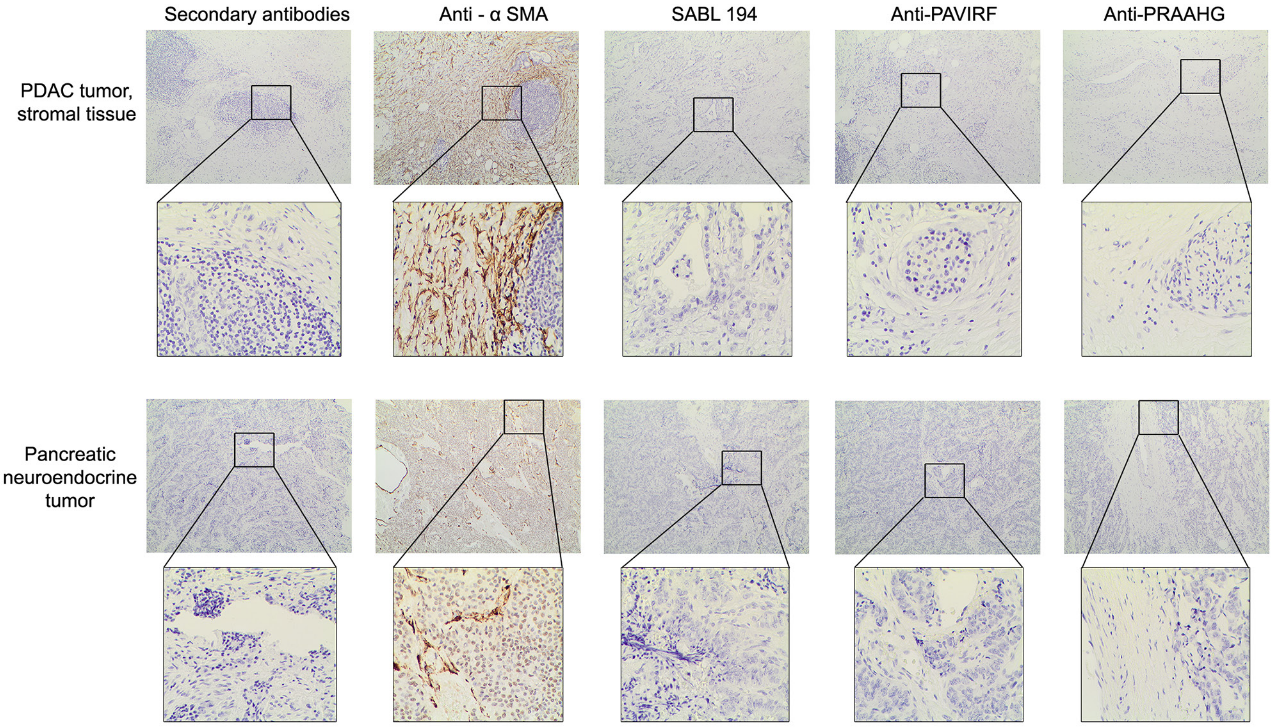

Supplementary Figure S3: Immunohistochemistry on control tissues. Formalin-fixed, paraffin-embedded tissue sections of the stromal area of a PDAC tumor or of a neuro-endocrine tumor were stained by anti-PAVIRF, anti-PRAAHG, SAB L194 and anti α-SMA antibodies. All immunohistochemistry steps were performed on the Leica BOND III automated system according to the manufacturers' instructions. Anti-peptide (anti-PAVIRF and anti-PRAAHG) and anti α-SMA antibodies were used at 0.2 μg/ml and 0.7 mg/ml, respectively. SAB L194 antibody was diluted at 1/1500 in Leica diluent.

Supplementary Table S1: Data collected on tumor tissue from patients with pancreatic ductal adenocarcinoma

Patients	Age[1]	Gender[2]	Tumor size (diameter[3])	TNM[4] T	N	M	Grade[5]	KRAS[6]	InsC [7] Repeat Number	Anti-PRAAGH reactivity
PDAC 1	59	0	4.5	2	1	0	2B	ND		+
PDAC 2	66	0	3.5	3	0	0	2A	ND		+
PDAC 3	54	0	3	3	1	0	4	G12D		+
PDAC 4	73	1	1.8	3	1	0	2B	ND		+
PDAC 5	62	0	2	3	1	0	2B	WT	9	++
PDAC 6	57	1	4.3	4	1	1	4	G12R	9	+++
PDAC 7	70	0	2.3	3	1	0	2B	G12V		ND
PDAC 8	63	1	2.5	3	1	0	2B	ND		=
PDAC 9	66	0	6	3	0	0	2A	ND		ND
PDAC 10	58	1	5.2	2	1	0	2B	G12V		-
PDAC 11	67	1	1.5	3	0	0	2A	G12D		++
PDAC 12	64	0	4.7	3	1	1	4	G12D		100% Stroma (-)
PDAC 13	57	1	6	3	1	0	2B	WT		100 % Stroma (-)
PDAC 14	63	1	4.5	3	0	0	2B	G12D		ND
PDAC 15	66	1	2.5	3	0	0	2A	ND		ND
PDAC 16	66	1	2.5	3	0	0	2A	G13R/D54Y		ND
PDAC 17	79	1	2	3	0	0	2A	ND		ND
PDAC 18	57	0	3	3	0	0	2A	G12V		++
PDAC 19	62	0	3	3	0	0	2A	ND		ND
PDAC 20	53	1	2	1	0	0	2A	Q61R		++
PDAC 21	61	1	2.3	3	1	0	2B	ND		ND
PDAC 22	72	0	3	3	1	0	2B	WT	9	+++
PDAC 23	69	1	3.5	3	0	0	2A	G12D		100 % Stroma (-)
PDAC 24	57	1	3.7	3	1	0	2B	G12D		-
PDAC 25	73	1	2	2	0	0	1B	ND		ND
PDAC 26	50	1	3	2	1	0	2B	ND		-
PDAC 27	87	1	3	3	1	0	2B	G12R		100 % Stroma (-)
PDAC 28	85	0	3.3	3	1	0	2B	WT		-
PDAC 29	79	1	3.5	3	1	0	2B	WT	4	+++
PDAC 30	73	1	4.1	3	1	0	2B	G12V		-
PDAC 31	81	0	2.4	3	1	0	2B	ND	9	+++
PDAC 32	76	0	NC	2	0	0	1B	ND	5	+++

[1]: in years. [2]: Male 0, Female 1.[3]: in centimeters. [4]: TNM stage: T: Tumor, N: Node, M: Metastasis. [5]: Tumor grade (WHO). [6] : genotyping performed on tissue after dissection of tumor tissue (see ref 31); [7]: DNA extracted from the whole tumoral tissue. ND = Not Disponible, WT = wild type, - = no reactivity, + = sparse reactivity (isolated cell); ++ = disseminated cell reactivity, +++ whole tissue reactivity.

Supplementary Table S2: Data collected on patients with non-malignant pancreatic diseases (non-MPD patients)

A : Detection of the InsC on DNA extracted from pancreatic tissue samples

Patients	Diseases	Age[1]	Gender[2]	InsC repeat number
non-MPD 1	IPMT	69	0	ND
non-MPD 2	IPMT	56	0	ND
non-MPD 3	CCP	61	0	8
non-MPD 4	CCP	72	0	ND
non-MPD 5	CCP	71	1	ND
non-MPD 6	RC	76	1	ND
non-MPD 7	CCP	40	1	ND
non-MPD 8	CCP	73	0	ND
non-MPD 9	CCP	53	1	ND
non-MPD 10	IPMT	74	1	ND
non-MPD 11	IPMT	71	0	ND

B : Detection of the InsC on DNA extracted from blood samples

Patients	Diseases	Age[1]	Gender[2]	InsC repeat number
non-MPD 12	CCP	48	0	ND
non-MPD 13	CCP	53	1	ND
non-MPD 14	CCP	54	0	ND
non-MPD 15	CCP	68	0	ND
non-MPD 16	CCP	45	0	ND
non-MPD 17	CCP	69	1	ND
non-MPD 18	CCP	43	0	ND
non-MPD 19	CCP	62	0	ND

ND = Not Detected, CCP = Calcifying Chronic Pancreatitis, IPMT = Intraductal Papillary Mucinous Tumor;, RC = Retention Cyst

[1] in years, [2]: Male 0, Female 1
